# Beyond blood brain barrier breakdown – *in vivo *detection of occult neuroinflammatory foci by magnetic nanoparticles in high field MRI

**DOI:** 10.1186/1742-2094-6-20

**Published:** 2009-08-06

**Authors:** Eva Tysiak, Patrick Asbach, Orhan Aktas, Helmar Waiczies, Maureen Smyth, Joerg Schnorr, Matthias Taupitz, Jens Wuerfel

**Affiliations:** 1 Cecilie Vogt Clinic for Neurology, Charité – University Medicine Berlin, Germany; 2 Department of Radiology, Charité – University Medicine Berlin, Campus Mitte, Germany; 3 Department of Neurology, Heinrich-Heine-University, Duesseldorf, Germany

## Abstract

**Background:**

Gadopentate dimeglumine (Gd-DTPA) enhanced magnetic resonance imaging (MRI) is widely applied for the visualization of blood brain barrier (BBB) breakdown in multiple sclerosis and its animal model, experimental autoimmune encephalomyelitis (EAE). Recently, the potential of magnetic nanoparticles to detect macrophage infiltration by MRI was demonstrated. We here investigated a new class of very small superparamagnetic iron oxide particles (VSOP) as novel contrast medium in murine adoptive-transfer EAE.

**Methods:**

EAE was induced in 17 mice via transfer of proteolipid protein specific T cells. MR images were obtained before and after application of Gd-DTPA and VSOP on a 7 Tesla rodent MR scanner. The enhancement pattern of the two contrast agents was compared, and correlated to histology, including Prussian Blue staining for VSOP detection and immunofluorescent staining against IBA-1 to identify macrophages/microglia.

**Results:**

Both contrast media depicted BBB breakdown in 42 lesions, although differing in plaques appearances and shapes. Furthermore, 13 lesions could be exclusively visualized by VSOP. In the subsequent histological analysis, VSOP was localized to microglia/macrophages, and also diffusely dispersed within the extracellular matrix.

**Conclusion:**

VSOP showed a higher sensitivity in detecting BBB alterations compared to Gd-DTPA enhanced MRI, providing complementary information of macrophage/microglia activity in inflammatory plaques that has not been visualized by conventional means.

## Background

A fundamental pathologic feature of multiple sclerosis (MS) is the formation of multifocal plaques in the central nervous system (CNS), accompanied by a disruption of the blood brain barrier (BBB). Gadopentate dimeglumine (Gd-DTPA) does not cross an intact BBB and can thus be used to detect BBB leakage in acute inflammatory lesions by Gd-DTPA enhanced MRI [1]. Recently, iron-oxide based magnetic nanoparticles have evolved as a new class of MRI contrast agents [2-6], bearing the potential to detect macrophage infiltrates into the CNS independently from BBB breakdown [7,8]. Macrophages play a pivotal role in the pathophysiology of MS, since they invade the CNS early during disease and act as effector cells in the inflammatory cascade, leading to persistent structural and functional tissue damage [9,10]. Dextran-coated magnetic nanoparticles have been applied in various animal models to visualize the migration of macrophages by MRI [2,7,8,11-15]. Two recent studies showed that the application of magnetic nanoparticles in MS patients resulted in a pattern that was distinct from BBB leakage visualized on Gd-DTPA enhanced images [16,17].

In this study, we investigated the capacity of novel, very small superparamagnetic iron oxide particles (VSOP) to detect neuroinflammatory foci in murine experimental autoimmune encephalomyelitis (EAE), an animal model of MS. VSOP are substantially smaller than conventional magnetic nanoparticles due to an electrostatically stabilized citrate coating [6], and therefore can also be used to detect BBB breakdown [18,19]. On the other hand, VSOP are very efficiently phagocytized [20] and were successfully applied for *in vivo *tracking of mononuclear cells in the past [21]. We analyzed the distribution pattern and kinetics of VSOP enhancement in adoptive-transfer EAE in comparison to conventional Gd-DTPA enhanced MRI and compared these findings with histopathological alterations.

## Materials and methods

### Adoptive-transfer EAE

Female SJL/J mice, six to eight weeks old, were purchased from Charles River (Sulzfeld, Germany). Animals were housed in sawdust-lined cages in a climate-controlled room and received standard rodent feed and water *ad libitum*. All experiments were approved by the local animal welfare committee and conformed to the European Communities Council Directive (86/609/EEC). For adoptive-transfer EAE, naïve donor mice were immunized with an emulsion containing 250 μg PLP (murine proteolipid peptide p139-151; purity > 95%, Pepceuticals, Leicester, UK) in equal volumes of phosphate buffered saline (PBS) and Complete Freund's Adjuvant (CFA, Difco Laboratories, Detroit, USA), and 4 mg/ml *Mycobacterium tuberculosis *H37Ra (Difco Laboratories, Detroit, USA), as previously described [22]. Ten days after immunization, cells were extracted from draining lymph nodes and restimulated with 12.5 μg PLP/ml in cell culture medium (RPMI 1640 supplemented with 2 mM L-glutamine, 100 U/ml penicillin, 100 μg/ml streptomycin and 10% fetal calf serum) for four days at 37°C. For adoptive transfer, 8–12 × 10^6 ^T-cell blasts in 100 μl PBS were injected intraperitoneally into 17 syngenic recipients.

Mice were weighed daily and scored for EAE [22]: 0, unaffected; 1, tail weakness or impaired righting on attempt to roll over; 2, paraparesis; 3, paraplegia; 4, paraplegia with forelimb weakness or complete paralysis; score > 4, to be sacrificed. Mice with a score of 4 received an intraperitoneal injection of 200 μl glucose (5%) daily.

### MRI analysis

Cerebral MRI was performed on a 7 Tesla rodent MRI scanner (Pharmascan 70/16AS, Bruker BioSpin, Ettlingen, Germany), applying a 20 mm RF-Quadrature-Volume head coil. Animals received anesthesia via facemask induced with 3% and maintained with 1.5 – 2.0% isoflurane (Forene, Abbot, Wiesbaden, Germany) delivered in 100% O_2 _under constant ventilation control (Bio Trig System, Bruker BioSpin, Ettlingen, Germany). Mice were placed on a heated circulating water blanket to keep up body temperature at 37°C.

Axial and coronal T1-weighted (MSME; TE 10.5 ms, TR 322 ms, 0.5 mm slice thickness, matrix 256 × 256, field of view (FOV) 2.8 cm, eight averages, 40 coronal slices, scan time 22 minutes, and 20 axial slices, scan time 16 min), fat-suppressed turbo spin echo T2-weighted (RARE; TE1 14.5 ms, TE2 65.5 ms, TR 4500 ms, 0.5 mm slice thickness, Matrix 256 × 256, FOV 2.8 cm, eight averages, 40 coronal slices, scan time 28 minutes, and 20 axial slices, scan time 28 minutes) and T2*-weighted (GEFI; TE 5.6 ms, TR 1200 ms, flip angle 35°, 0.5 mm slice thickness, Matrix 256 × 256, FOV 2.8 cm, four averages, 40 coronal slices, scan time 20 minutes, and 20 axial slices, scan time 13 minutes) images were acquired before and after intravenous (i.v.) administration of the respective contrast agent. Identical slice positions were used for all sequences applied: coronal slices were aligned to the olfactory bulb/frontal lobe fissure and covered the entire brain up to the cervical spinal cord. Axial slices were positioned parallel to a plane through the most frontal tip of the olfactory bulb and the most rostral cerebellar part. MRI data were analyzed using the MEDx3.4.3 software package (Medical Numerics, Virginia, USA) on a LINUX workstation.

Mice were investigated daily for the development of BBB breakdown beginning on the fifth day post T cell transfer with MRI immediately after injection of 0.2 mmol/kg bodyweight Gd-DTPA (Magnevist, Bayer-Schering Pharma AG, Berlin, Germany) into the tail vein. If parenchymal contrast enhancement was detected, animals received 0.2 mmol/kg bodyweight VSOP (VSOP C-184, Ferropharm, Teltow, Germany) and MR investigations were repeated after 24 h. Three animals with rapid onset received primarily VSOP and no Gd-DTPA.

### Histology

For subsequent histological analysis, mice were lethally anaesthetized (Xylazinhydrochlorid, Rompun 2%, Bayer, Leverkusen, Germany, and Ketamin, CuraMED Pharma, Karlsruhe, Germany) and intracardially perfused with 0.1 M PBS and fixed in 4% paraformaldehyde (PFA) in 0.1 M PBS. Brain and spinal cord were removed and postfixed in 4% PFA. The tissue was then dehydrated in 30% sucrose for cryoprotection and stored at -80°C. Axial cryosections (Jung cryostat 2800 Frigocut-E, Cambridge Instruments, Nussloch, Germany) of the entire brain were stained either by standard hematoxylin and eosin (H&E) procedure to detect cellular inflammation or by Prussian Blue staining according to Perl's method [23] to detect VSOP.

For further characterisation of microglia/macrophages, tissue sections were incubated with rabbit anti-IBA-1 antibodies (1:1000, Wako Chemicals, Neuss, Germany) in PBS overnight at 4°C after preincubation in 10% normal goat serum (CALTAG, Invitrogen, Karlsruhe, Germany) to block non-specific binding, and then with a secondary antibody (anti-rabbit Cy2, Amersham, Munich, Germany) for 1 h at room temperature. Slices were counterstained with Hoechst-33258 (Molecular Probes, Leiden, the Netherlands) to visualize cell nuclei and with rhodamine phalloidin (Molecular Probes, Leiden, the Netherlands) to identify vascular structures. Finally, all slices were washed three times in PBS and cover-sealed with fluorescence mounting medium (DAKO Deutschland GmbH, Germany). Selected sections were examined by epifluorescence microscopy and digitally photographed (Olympus BX-51, Hamburg, Germany). Images were assembled using Adobe Photoshop (Adobe Systems, San Jose, CA, USA).

## Results

### Clinical EAE course

**Figure 1:**
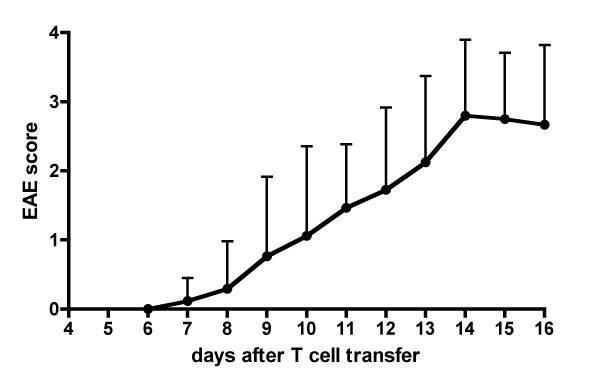
**Clinical disease course**. Clinical disease course in 17 mice after transfer (day 0) of proteolipid protein specific encephalitogenic T cells. Mean experimental autoimmune encephalomyelitis (EAE) scores and standard deviations are presented. Two animals were followed up until complete recovery (data for extended observation phase not shown).

All mice developed EAE, presenting first clinical symptoms 7 – 14 (mean 9.9) days after the transfer of encephalitogenic T cells, most commonly initiated by an impaired tail motility and a delayed righting reflex after the animal was rolled on its back. The disease severity progressed to peak EAE scores between 1 and 4 (mean 2.5 ± 1.1) within several days, as illustrated in figure 1. Two animals were followed up longitudinally for three weeks until complete recovery.

### VSOP enhanced MRI visualized inflammatory foci beyond BBB breakdown depicted by Gd-DTPA

**Figure 2:**
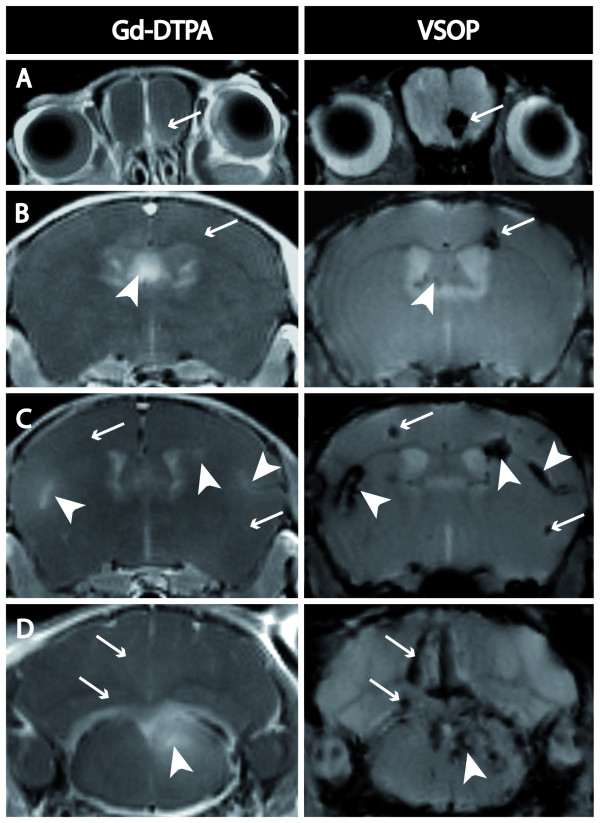
**Comparison of contrast enhancing lesions**. Comparison of coronal T1-weighted images immediately after Gd-DTPA administration versus T2*-weighted images 24 h after VSOP application. The majority of the lesions enhanced both, Gd-DTPA and VSOP, although shape differences within individual lesions were apparent (arrow heads). Furthermore, several plaques enhanced exclusively VSOP, but not Gd-DTPA (small arrows).

Contrast enhanced MRI visualized 55 inflammatory plaques, most commonly in the brain stem and the periventricular area, but also disseminated throughout the remaining CNS (table 1). Areas of BBB breakdown appeared hyperintense on T1-weighted images after Gd-DTPA application or, respectively, hypointense on T2*-weighted images after VSOP enhancement (figure 2). The majority of the *in vivo *detected plaques were enhanced by both contrast agents (38 lesions), nevertheless, the enhancement pattern of Gd-DTPA and VSOP differed within the individual lesion, as demonstrated in figure 2 (arrow heads). In general, Gd-DTPA enhanced lesions appeared more diffusely, gradually fading towards their edges, whereas VSOP hypointensities presented concise hypointense spots with clear margins towards the surrounding tissue.

**Table 1:** Distribution and number of contrast enhancing lesions (CEL).

Brain region	Exclusively VSOP enhancement	Gd-DTPA and VSOP enhancement	Total number of CEL
Brain stem	+++	+++++++++	12
Periventricular	++	++++++++++	12
Midbrain		+++++++++	9
Cerebellum	++	++++++	8
Olfactory bulb	+++	+++++	8
Cortex	+++	+++	6

	13	42	55

Data are given from 14 mice after Gd-DTPA and VSOP administration, respectively. Three mice received VSOP only and are not included in the table. Each + refers to an individual animal.

Of note, 13 out of 55 inflammatory foci were exclusively visualized by VSOP, but could not be detected on Gd-DTPA enhanced T1-weighted images (table 1). Although these lesions did not show the conventional BBB breakdown characteristic defined by Gd-DTPA enhancement, they did not principally differ in terms of temporal or spatial MRI appearance in this study. Various examples are presented in figure 2 (small arrows). Vice versa, every Gd-DTPA enhancing lesions also clearly delineated by VSOP.

### Histological correlation

**Figure 3:**
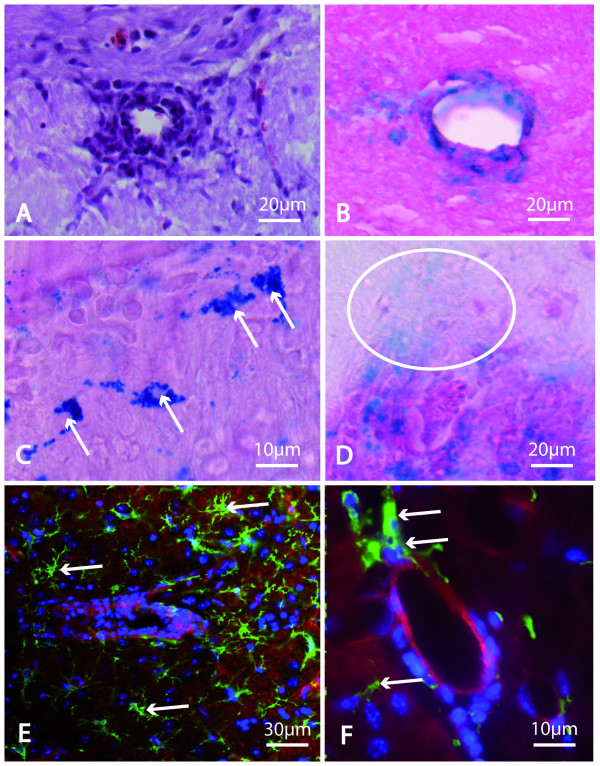
**Histological findings**. EAE-typical perivascular cell infiltrations were identified on Hematoxylin & Eosin stained slices (A). After Prussian Blue staining, VSOP was detected on corresponding sites (B). Two different distribution patterns of magnetic nanoparticles became evident: The incorporation of VSOP into cytoplasmatic vesicles within phagocytic cells (arrows in C), and a diffuse accumulation in the brain parenchyma (ellipse in D). IBA-1 positive macrophages/microglia were identified by immunofluorescent staining within perivascular plaques, colocalizing with Prussian Blue positive cells (arrows in E, and in higher magnification in F; green: anti-IBA-1, macrophages/microglia; blue: Hoechst 33258, cell nuclei; red: rhodamin phalloidin, vascular structures).

Each Gd-DTPA or VSOP enhancing MRI lesion showed tissue pathology also on the corresponding histological slices. Perivascular and perimeningeal cell infiltrations were clearly demarked in H&E stainings (figure 3A). On Prussian Blue stained slices, iron depositions could be co-localized to these areas (figure 3B). VSOP was visualized within cellular compartments, suggesting their incorporation into cytoplasmatic vesicles (figure 3C). Furthermore, we detected extracellular magnetic nanoparticles accumulating diffusely within the brain parenchyma (figure 3D). Immunofluoresence stainings identified IBA-1 positive microglia/macrophages within perivascular lesions, which could be co-localized by their morphology to those cells exhibiting intravesicular VSOP on Prussian blue stainings (figure 3E,F).

### Kinetics and characterization of VSOP contrast

**Figure 4:**
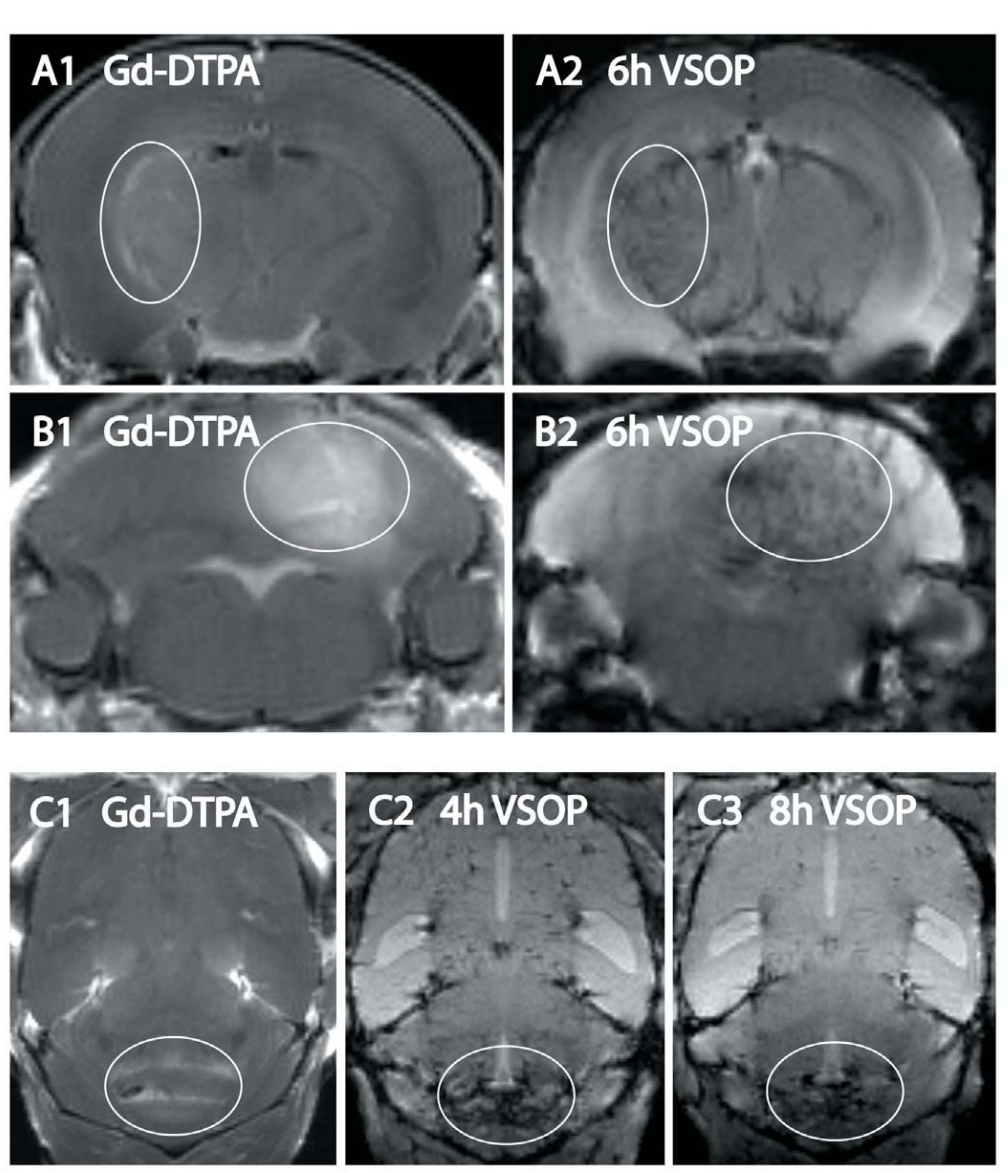
**Early VSOP enhancement**. In regions of blood brain barrier breakdown visualized by Gd-DTPA (A1, B1, C1), parenchymal VSOP leakage was detectable within the initial 6 h post application (A2, B2). Lesions became clearly distinguishable from the vasculature only after the blood pool contrast resolved. In C2, 4 h post application, intravascular VSOP is still visible. Eight hours after application, neuroinflammatory foci remain hypointense, where as intravascular signal decrease vanished (C3). Ellipses highlight contrast enhancing lesions.

**Figure 5:**
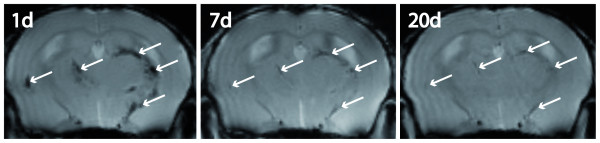
**Time course of VSOP enhancement**. In a longitudinal follow-up, T2*-hypointense lesions (arrows) were depicted at 24 h, 7 days and 20 days post VSOP application. Some hypointense plaques remained visible after 20 days.

VSOP were strongly prominent in the blood pool as well as in areas of BBB breakdown within the first 6 h after application (figure 4). Figure 4 as an example depicts the typical signal void of inflammatory lesions 4–8 h post VSOP application in the diencephalon (A) and the cerebellum (B, C). In these early time points, regions with uniform Gd-DTPA enhancement did not appear accordingly evenly hypointense on co-localized T2*-weighted images after VSOP application, but these lesions were depicted clear-cut and more widespread in comparison (figure 4A, B). At later time points, i.e. after 18–24 h, parenchymal VSOP enhancement became most pronounced, when the non-specific blood pool contrast had completely vanished, and gradually ceased thereafter. Remarkably, some hypointense spots were still detectable 20 days after the initial application, despite complete clinical recovery of the animals at this time point (figure 5).

**Figure 6:**
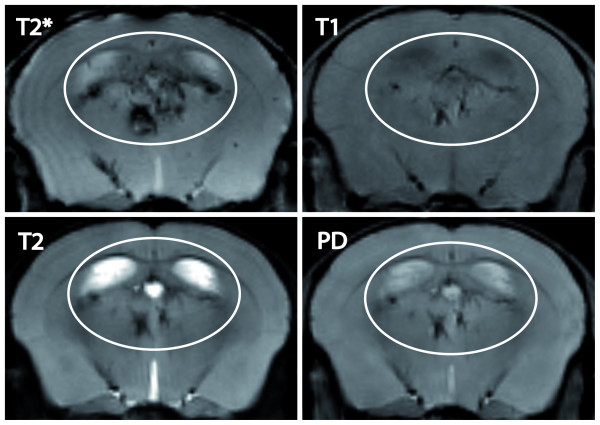
**VSOP contrast in different MR sequences**. Prominent contrast-enhancing periventricular lesions are depicted 24 h after VSOP injection on coregistered coronal T2*-, T2-, T1- and proton-density-weighted images (ellipse). T2*-weighted MRI was most sensitive in detecting magnetic nanoparticles. On T1-weighted images, very high VSOP concentrations became visible as hypointense spots at 7 Tesla.

T2*-weighted sequences were most sensitive for the detection of magnetic nanoparticles, as illustrated in figure 6. None of the lesions could be differentiated prior to contrast agent administration in any of the investigated MRI sequences (T1-, T2-, T2*- or proton-density-weighted MRI), nor were lesions detectable in healthy control mice.

## Discussion

In this study we investigated the capability of novel electrostatically stabilized magnetic nanoparticles, VSOP, to detect neuroinflammatory foci in murine adoptive transfer EAE, a disease model of MS. Although MRI revolutionized the diagnosis and management of MS patients [24], a persistent mismatch between clinical and MRI findings has remained [25,26]. Conventional MRI depicted hyperintense lesions on T2-weighted MRI as relatively unspecific traces of the disease and showed evidence of acutely disrupted BBB indicated by Gd-DTPA leakage into the parenchyma [27]. Both conventional imaging techniques correlated only weakly or moderately to disability and clinical outcome of our patients [28]. Experimental contrast agents such as Gadofluorine M revealed blood brain barrier breakdown with higher sensitivity than conventional Gd-DTPA, but have not been applied to humans [18,29]. A new class of MRI contrast agents based on superparamagnetic iron oxide cores was recently developed and originally employed to visualize labeled cells [5,30-32]. In EAE, macrophages could be detected *in vivo *within inflammatory lesions after phagocytosis of magnetic nanoparticles [8,12,15,18,33]. Our finding of intracellular iron oxide, verified by Prussian Blue stained histology, is in line with these reports. Recent studies applying larger dextran-coated magnetic nanoparticles report their detection on MRI after a time delay of 24 h, presumably depending on the invasion of macrophages rather than on magnetic nanoparticles passively diffusing through the disrupted BBB barrier [4,8,12].

However, the extremely small VSOP investigated in this study became immediately visible after application as prominent T2*-hypointensity. Generally, VSOP detected lesions were in good spatial agreement to those areas that enhanced after Gd-DTPA application on T1-weighted MRI. Nevertheless, VSOP caused very distinct hypointense spots, whereas Gd-DTPA enhanced lesions appeared less clear cut on T1-weighted images. Most intriguingly, a subset of 13 out of 55 lesions became visible exclusively after VSOP injection, remaining unenhanced after concomitant Gd-DTPA application. In our study, these "VSOP-only" lesions did neither principally differ in space nor in time from those inflammatory plaques that simultaneously enhanced both contrast agents, or on corresponding histology. Prussian-blue positive magnetic nanoparticles were present in vesicles within cells that were identified as macrophages/microglia on immunofluorescent stainings, and also as a diffuse extracellular accumulation within the connective tissue in vicinity to inflammatory plaques. Thus, VSOP enhanced MRI was capable of visualizing both, BBB disruption at high sensitivity as well as macrophage infiltration into neuroinflammatory lesions.

The occurrence of extracellular VSOP deposits in histology detected in regions without Gd-DTPA enhancement appears inscrutable at first sight. Two different underlying mechanisms have to be considered. First, BBB breakdown might be present but very subtle in these regions. Paramagnetic nanoparticles such as VSOP are characterized by a strong susceptibility effect in T2*-weighted imaging, possibly providing an augmented sensitivity for BBB alterations compared to Gd-DTPA. Secondly, presuming BBB integrity, VSOP might also accumulate in inflammatory foci due to a locally enhanced activity of transendothelial transport mechanisms during inflammation [34,35]. Nanoparticles were shown to facilitate drug delivery across the BBB [36]. Transport of transferrin into the CNS occurs via receptor-mediated transcytosis [37]. Which particular pathways might channel VSOP across an intact BBB remains to be determined.

Magnetic nanoparticles have already been applied in several human trials of cerebral ischemia [38,39] and in brain tumours [40]. In MS, two studies on magnetic nanoparticles were reported so far: In a prospective trial of Dousset et al. comprising ten MS patients with relapsing-remitting disease course, two patients presented enhancing lesions exclusively after administration of magnetic nanoparticles [17]. A very recent study by Vellinga et al. reported a mismatch of 144 out of 188 lesions in 14 patients, that could be visualized by dextran-coated magnetic nanoparticles (USPIO) enhanced MRI, but not by Gd-DTPA. Vice versa, of 59 Gd-DTPA positive lesions, 15 were USPIO negative in the same study [16]. Despite these encouraging initial experimental human trials, the exact specificity and sensitivity of different magnetic nanoparticles applied remains to be elucidated, since they obviously depict different aspects of pathology, as indicated by the data presented in this study.

VSOP has been investigated as a blood pool contrast agent in MR-angiography of the coronary arteries in a clinical phase 1B study, and was well tolerated [41]. Therefore, a future application also in MS seems feasible.

## Conclusion

Here, we demonstrated that VSOP, due to their small size and special surface characteristics, uniquely combine the advantages of an improved detection of occult BBB alterations, adding the capability of visualizing subtle macrophage infiltration into active neuroinflammatory plaques. Moreover, VSOP differ from other magnetic nanoparticles in their capability to exclusively image subtle BBB disruptions. Several lesions could be detected that enhanced solely VSOP, indicating the sensitivity for additional neuroinflammatory processes so far missed by conventional contrast media. Thus, novel magnetic nanoparticles may contribute to resolve the clinico-radiological paradox in future human trials.

## Abbreviations

BBB: blood brain barrier; CNS: central nervous system; Gd-DPTA: gadopentate dimeglumine; MRI: magnetic resonance imaging; MS: multiple sclerosis; PBS: phosphate buffered saline; PFA: paraformaldehyde; USPIO: ultrasmall superparamagnetic iron oxide particles; VSOP: very small superparamagnetic nanoparticles.

## Competing interests

The authors declare that they have no competing interests.

## Authors' contributions

ET carried out animal experiments, histological analysis, MR imaging, and drafted the manuscript. PA established the MR imaging and revised the manuscript. OA instructed the animal experiments and revised the manuscript. MS participated in the animal experiments and MR imaging. JS designed and provided the contrast media and revised the manuscript. MT supervised MR imaging and revised the manuscript. JW designed the study, carried out the MR imaging and drafted the manuscript. All authors read and approved the final manuscript.
